# Uric Acid in Primary Hyperparathyroidism: Marker, Consequence, or Bystander?

**DOI:** 10.3390/metabo15070444

**Published:** 2025-07-02

**Authors:** Matteo Malagrinò, Guido Zavatta

**Affiliations:** 1 Division of Endocrinology and Diabetes Prevention and Care, IRCCS Azienda Ospedaliero-Universitaria di Bologna, 40138 Bologna, Italy; matteo.malagrino2@unibo.it; 2 Department of Medical and Surgical Sciences (DIMEC), Alma Mater Studiorum University of Bologna, 40138 Bologna, Italy

**Keywords:** primary hyperparathyroidism, uric acid, vitamin D, bone, kidney, gut, ABCG2, cardiovascular, chronic kidney disease, guidelines

## Abstract

**Background:** Several recent studies have documented an increased cardiovascular risk in patients with primary hyperparathyroidism (PHPT), thereby stimulating interest in the association with uric acid (UA), a metabolite linked to cardiovascular disease and chronic kidney disease (CKD) progression, whose role in these conditions is still the subject of study. The aim of this review is to summarize the underlying pathophysiological mechanisms of the PHPT-UA relation and discuss their potential clinical implications. **Methods:** We conducted a comprehensive literature review, with a focus on the physiological and clinical aspects of the relationship between PHPT and UA. **Results:** The evidence in the literature supports the association between PHPT and elevated UA levels, although the underlying mechanisms still need to be elucidated. Key mechanisms seem to involve tubular and intestinal transporters, particularly the ABCG2 transporter, as well as indirect effects mediated by hypercalcemia and inflammatory processes. **Conclusions:** The association between PHPT and UA, though recognized for years, highlights the existence of linked pathophysiological mechanisms between mineral and purine metabolism. However, the current knowledge does not clarify whether uric acid plays an active role in the development of complications related to hyperparathyroidism or if it just represents an indirect marker of metabolic dysfunction. In the absence of specific guidelines, measuring UA levels to screen for hyperuricemia, especially in patients with additional risk factors, should be considered to prevent related complications. Future studies could clarify the role of UA in PHPT, improving our understanding of the disease and potentially leading to new therapeutic strategies to prevent cardiovascular, renal and joint manifestations.

## 1. Introduction

Primary hyperparathyroidism (PHPT) is a common endocrine disorder of calcium metabolism characterized by hypercalcemia and elevated or inappropriately normal concentrations of parathyroid hormone (PTH) [1] due to a benign overgrowth of parathyroid tissue either as a single-gland (80% of cases) or as a multiple-gland disorder (15–20% of cases). In recent years, PHPT has been identified with increasing frequency in an asymptomatic phase, often detected incidentally during routine blood tests or as part of an osteoporosis evaluation [2]. However, the presence of target organ damage, including osteoporosis, kidney stones, and a reduced eGFR, is already present.

Recent studies have documented an elevated cardiovascular risk in patients with PHPT, thereby stimulating a growing interest in the association between PHPT and uric acid (UA), a metabolite whose links to cardiovascular disease and chronic kidney disease (CKD) progression are well-documented and still under study [3,4,5,6]. The relationship between UA and PTH has been recognized for many years [7,8], and substantial evidence supports this association in primary hyperparathyroidism [9], secondary hyperparathyroidism [10], and even in the general population [11]. A multitude of mechanisms have been postulated to elucidate this association. The most compelling hypothesis involves the action of PTH on tubular UA transporters [12]. However, other potential mechanisms may also play a role, such as mild systemic inflammation, which has been described in both hyperparathyroidism and hyperuricemia [13,14], or the direct involvement of hypercalcemia, regardless of PTH [9,15,16]. Despite these findings, in the current guidelines for the management of PHPT, an evaluation of UA levels is not recommended for these patients [17,18,19]. This review aims to summarize the extant evidence on the association between PHPT and UA metabolism, explore the underlying pathophysiological mechanisms, and discuss the potential clinical consequences.

## 2. Uric Acid Metabolism, Excretion, and Definition of Hyperuricemia

### 2.1. Uric Acid Metabolism and Excretion

Uric acid is a heterocyclic organic compound formed by purine catabolism, in particular adenine and guanine [20]. While synthesis and purine degradation occur in all tissues, the production of UA is restricted to organs where the enzyme xanthine oxidase is present, primarily in the liver and small intestine. In humans, the evolutionary loss of the uricase enzyme has led to the absence of further degradation of UA, whereas other mammals have retained this enzyme, resulting in the conversion of UA to allantoin, a more soluble compound [21]. Urate, the ionized form of UA, is predominantly found in plasma as monosodium urate (MSU), which reaches the saturation point at a serum concentration of 6.8 mg/dL. Above this value, at serum pH 7.4, MSU tends to precipitate, forming crystals. Urate production depends on introducing purine in the diet, the amount of endogenous purine synthesis de novo, and the recycling of purine precursors by the enzyme phosphoribosyltransferase.

The kidneys are responsible for the majority of UA excretion, filtering out approximately 70% of the UA produced daily, while the remaining 30% is excreted from the intestine [4]. The kidney excretion process is complex and involves the distinct phases of glomerular filtration, tubular reabsorption, secretion, and post-secretory reabsorption. Only a minimal quantity of the filtered UA, approximately 8–12%, is excreted into the urine [22]. The transporters URAT 1 (SLC22A12) and GLUT9 (SLC2A9) play key roles in UA reabsorption in the proximal tubule. URAT1 mediates UA reabsorption from the tubular lumen into epithelial cells, while GLUT9 promotes UA transport through the basolateral membrane of tubular cells [22,23].

There are also proteins responsible for UA secretion in the tubular lumen, such as OAT1/3, which are located in the basolateral membrane of tubular cells, and ABCG2, an efflux transporter located in the apical membrane of epithelial cells, which consume the energy derived from the hydrolysis of ATP to carry metabolites, such as UA, into the tubular lumen [24,25].

**Figure 1:**
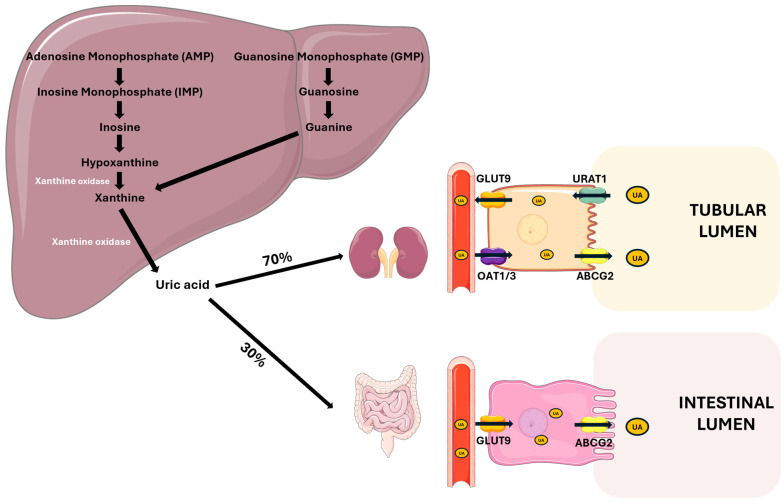
**Uric acid metabolism and excretion.** UA is primarily synthesized in the liver from the purine bases adenine and guanine through a series of enzymatic steps. Xanthine oxidase plays a central role by catalyzing the oxidation of hypoxanthine to xanthine, and xanthine to UA. Approximately 70% of UA is excreted by the kidneys through filtration, reabsorption, and secretion processes. The main renal tubular transporters involved are GLUT9 and OAT1/3 on the basolateral membrane and URAT1 and ABCG2 on the apical membrane. The remaining 30% is eliminated via the intestine, involving GLUT9 for basolateral uptake and ABCG2 for luminal secretion. Abbreviations: UA, uric acid; GLUT9, glucose transporter 9; OAT1/3, organic anion transporter 1/3; URAT1, urate anion transporter 1; ABCG2, ATP-binding cassette super-family G member 2. Adapted from Fiori et al. [28].

ABCG2 is also one of the main excretion mechanisms at the intestinal level, where it is responsible for UA secretion in the intestinal lumen. Several studies have demonstrated that ABCG2 dysfunction is associated with higher UA serum levels, highlighting its role in UA excretion [26,27] (Figure 1).

### 2.2. Definiton of Hyperuricemia

Hyperuricemia occurs under conditions of higher UA production due to increased dietary intake or accelerated cellular turnover and reduced UA excretion because of either impaired renal or intestinal excretion.

The exact definition of hyperuricemia is also debated. For many years, the biological saturation threshold of 6.8–7 mg/dL has been proposed, above which monosodium urate (MSU) crystals begin to precipitate in body fluids [29]. However, depending on the context, “epidemiological cutoffs” have been proposed based on studies associating hyperuricemia with poorer cardiovascular and renal outcomes. Some of these cutoffs are sex-specific, considering the uricosuric effects of estrogens and higher lean mass (thus higher nucleotide turnover) in men [21,30].

Recently, the SIMETAP-HU study confronted the use of two different diagnostic criteria to define hyperuricemia in order to homogenize the cutoffs used in the literature: the first one was based on epidemiological evidence (UA ≥ 7.0 mg/dL for men and ≥6.0 mg/dL for women) and the second one was based on physiochemical evidence (UA ≥ 7.0 mg/dL for both sexes). The study found that the associations of cardiovascular, renal, and metabolic factors with hyperuricemia diagnosed according to physiochemical criteria were more similar between men and women than those using epidemiological criteria. Therefore, the study proposes using a single 7 mg/dL cutoff.

### 2.3. Clinical Manifestations Associated with Hyperuricemia

From the clinical perspective, manifestations associated with hyperuricemia are linked to the ectopic deposition of MSU crystals in the joints, where they cause a chronic arthritis known as gout, and in the kidneys, where it can induce chronic interstitial nephropathy, kidney stones, and renal fibrosis, determining the conditions known as gouty nephropathy.

In recent years, these “traditional manifestations” have become less frequent due to the earlier diagnosis of hyperuricemia and the availability of urate-lowering drugs. As a result, the focus has shifted to the roles of UA in long-term cardiovascular and kidney damage.

Elevated UA levels have been recognized as contributing to the development of cardiovascular disease through mechanisms such as oxidative stress, endothelial dysfunction, systemic inflammation, and activation of the renin–angiotensin system, all of which contribute to vascular injury and remodeling [31,32]. Hyperuricemia and insulin resistance are also closely linked. UA-induced inflammation can make tissue insulin-resistant by reducing nitric oxide bioavailability in endothelial cells and interfering with insulin signaling pathways (IRS1/PI3K/Akt) [33,34]. On the other hand, insulin resistance seems to enhance the activity of the tubular transporter responsible for reabsorbing urine, thereby reducing its excretion [35,36]. Several studies have demonstrated an association between hyperuricemia and a greater incidence of hypertension, carotid atherosclerosis, and ischemic heart disease, and hyperuricemia has been identified as an independent predictor of total and cardiovascular mortality [5,37,38,39,40].

Nevertheless, it is still necessary to clarify whether hyperuricemia represents a biomarker of metabolic syndrome and kidney dysfunction or if it plays a causal role in target organ damage, as well as the efficacy of urate-lowering drugs, such as allopurinol and febuxostat, in preventing cardiovascular events and CKD progression [28,41].

## 3. Primary Hyperparathyroidism

### 3.1. Primary Hyperparathyroidism Definition and PTH Actions

Hypercalcemic PHPT is defined by the finding of an elevated serum calcium level adjusted for albumin in the presence of an elevated or inappropriately normal intact PTH level (using either a second or third-generation assay) on two occasions at least two weeks apart [17]. The excessive secretion of PTH by one or more parathyroid glands is usually caused by an adenoma or polyglandular hyperplasia or, less commonly, by carcinoma of the parathyroid glands [42,43].

From a physiological perspective, PTH acts on three target tissues to maintain calcium and phosphate homeostasis.

#### 3.1.1. PTH Actions in the Kidney

**Figure 2:**
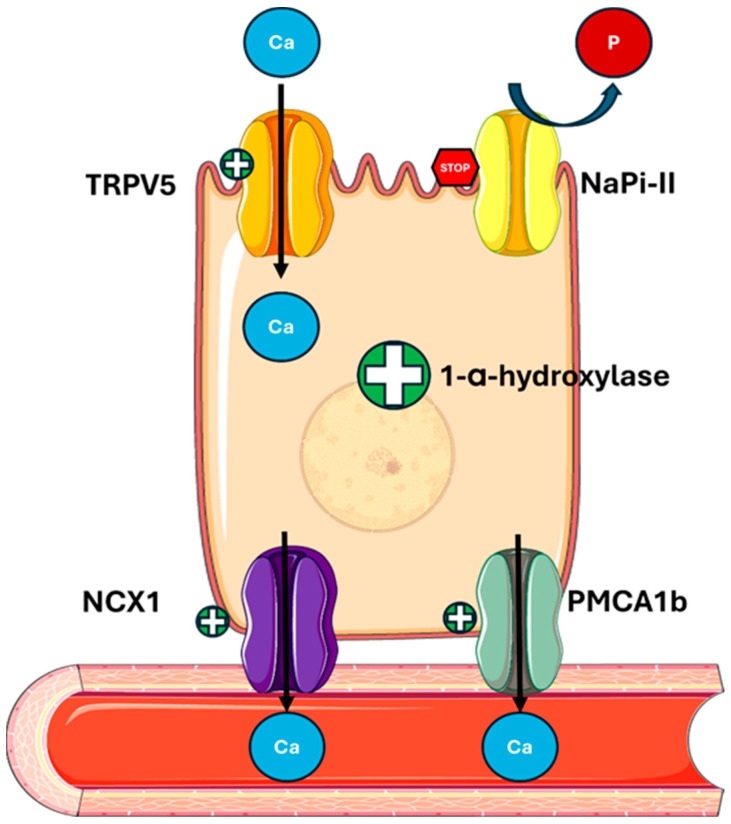
**PTH actions on the kidney.** PTH regulates mineral metabolism by stimulating calcium reabsorption through the upregulation of TRPV5 on the apical membrane and NCX1 and PMCA1b on the basolateral membrane of tubular cells. It also decreases phosphate reabsorption by downregulating NaPi-II cotransporters and enhances 1-α-hydroxylase activity, increasing the conversion of 25(OH)D to active 1,25(OH)_2_D. Abbreviations: Ca, calcium; P, phosphate; TRPV5, transient receptor potential cation channel subfamily V member 5; NaPi-II, sodium-dependent phosphate transport protein 2; NCX1, sodium/calcium exchanger; PMCA1b, plasma membrane calcium ATPase.

In the kidney, PTH stimulates calcium reabsorption and phosphate loss (Figure 2) by

- Promoting the expression and activity of apical channel TRPV5 (transient receptor potential vanilloid member 5), increasing calcium entry into tubular cells;
- Increasing the expression of the basolateral transporter NCX1 (sodium-calcium exchanger) and PMCA1b (plasma membrane calcium ATPase), facilitating the transfer of calcium into the bloodstream [44,45];
- Regulating Claudin-14, a tight junction protein, involved in calcium paracellular transport in the ascending limb of Henle’s loop (TAL) [46];
- Reducing sodium reabsorption by the Na/Cl cotransporter (NCC) in the proximal tubule, indirectly promoting the TRPV5 activity [47];
- Downregulating the sodium–phosphate cotransporters NaPi-IIa (SLC34A1) and NaPi-IIc (SLC34A3) in the proximal tubule, thus increasing phosphate excretion and altering the function of Pit-2 (SLC20A2), a less-known phosphate transporter [48,49,50].

#### 3.1.2. PTH Actions in Bone

**Figure 3:**
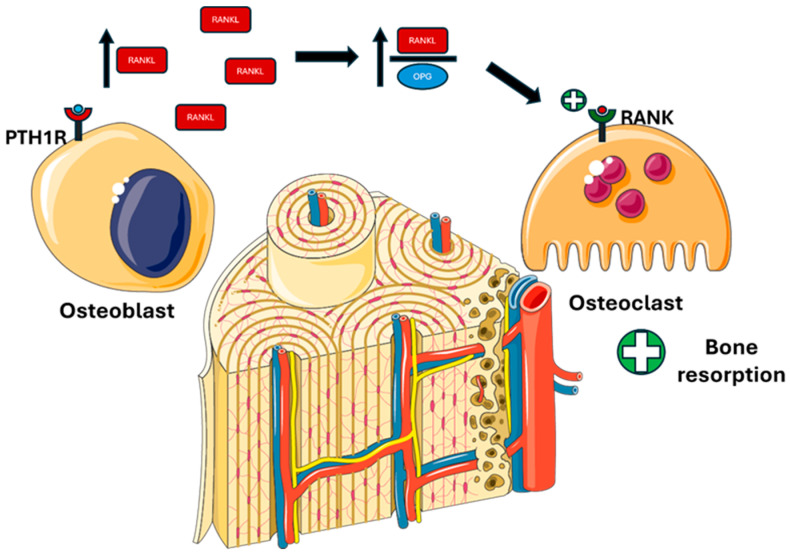
**PTH actions on bone.** PTH binds to PTH1R on osteoblastic cells, promoting RANKL synthesis and inhibiting OPG, thereby increasing the RANKL/OPG ratio and enhancing osteoclast differentiation and activity. Abbreviations: PTH1R, parathyroid hormone 1 receptor; RANKL, receptor activator of nuclear factor kappa-Β ligand; OPG, osteoprotegerin; RANK, receptor activator of nuclear factor κB.

In bone, PTH stimulates bone resorption, inducing calcium and phosphate mobilization (Figure 3).

- Activating PTH1R on the osteoblastic surface stimulates RANKL (receptor activator of nuclear factor kappa-Β ligand) production and inhibits OPG (osteoprotegerin), thereby stimulating differentiation and osteoclastic activity [51].

#### 3.1.3. PTH Actions in the Gut

**Figure 4:**
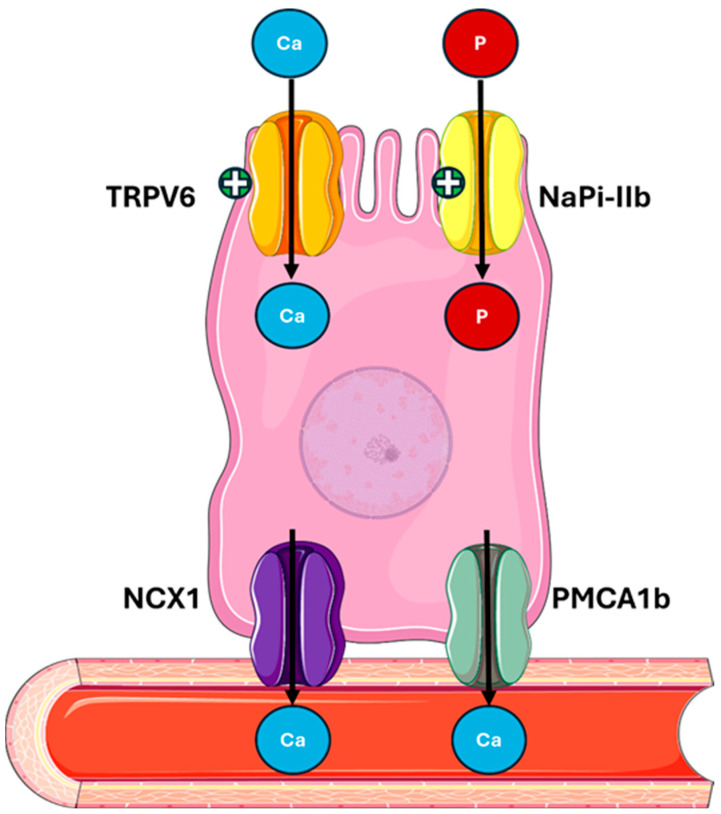
**PTH actions on the gut mediated by 1,25(OH)_2_D.** PTH indirectly stimulates the activation of vitamin D, which enhances calcium absorption via TRPV6, NCX1, and PMCA1b, and phosphate absorption via NaPi-IIb in the intestine. Abbreviations: Ca, calcium; P, phosphate; TRPV6, transient receptor potential cation channel subfamily V member 6; NaPi-IIb, sodium-dependent phosphate transport protein 2B; NCX1, sodium/calcium exchanger; PMCA1b, plasma membrane calcium ATPase.

In the gut, PTH acts indirectly by stimulating the production of active vitamin D 1,25(OH)_2_D, which enhances calcium and phosphate absorption (Figure 4).

- Regulating the expression of protein calcium transport proteins, such as TRPV6 (transient receptor potential vanilloid subfamily member 6) and calbindin-D9k [52,53];
- Increasing the expression of the sodium–phosphate cotransporter NaPi-IIb (Sodium-dependent phosphate transport protein IIB) in the brush border of intestinal cells [53,54].

### 3.2. Clinical Manifestation Associated with Primary Hyperparathyroidism

From a clinical perspective, in resource-rich health care systems, less than 20% of patients present with overt symptoms [55]. The onset of classical severe hypercalcemia, such as mental dullness or neuromuscular weakness, is today very rare and often related to rare cases of parathyroid carcinoma because the diagnosis of PHPT occurs earlier in asymptomatic stages, when calcium is measured during routine blood tests or assessments for osteoporosis. The main clinical complications of PHPT are nowadays osteoporosis, kidney stones, and progressive renal impairment. An increased incidence of gastrointestinal symptoms such as constipation and neuropsychiatric manifestations like fatigue, depression, and memory impairment is observed, although the causal link with PHPT is uncertain [55,56,57].

Among the lesser-known manifestations of PHPT are conditions such as calcium pyrophosphate crystal deposition disease [58,59,60,61,62], a prevalent joint manifestation, and gout [63,64]. However, documented cases of the latter remain comparatively limited.

In recent years, there has also been a growing interest in cardiovascular complications related to PHPT, and increased risks of hypertension, arrhythmia, left ventricular hypertrophy (LVH), valvular and myocardial calcifications, and cardiovascular mortality have been documented [55,65,66].

## 4. Clinical Evidence of the Association Between Primary Hyperparathyroidism and Uric Acid

The association between PHPT and UA has been documented in the literature for many years. As early as 1964, Scott et al. reported a case series of 12 patients affected by parathyroid adenoma that presented as elevated UA levels preoperatively. Of these, eleven (91.7%) patients were hyperuricemic, and five (45%) had clinically manifested gout [8]. Clearly, PHPT presentations were different in those years, with patients presenting with much higher calcium levels at diagnosis than we observe today.

Hui et al. examined the relationship between PTH and UA in 8316 individuals representing the US population using data from the National Health and Nutrition Examination Survey (NHANES) [11]. They divided the population into quintiles based on serum PTH levels and observed a significant increase in the UA level with each quintile. Even after adjusting the data for major confounding factors, such as age, sex, body mass index (BMI), diet, renal function, calcium, phosphorus, alkaline phosphatase, and vitamin D, they found a mean difference in the UA level of 19 μmol/L (0.32 mg/dL) between the first and fifth PTH quintiles (95% CI: 12–26 μmol/L; *p* < 0.001). Furthermore, comparing the highest and the lowest quintiles revealed an odds ratio of 1.39 (95% CI: 1.03–1.88; *p* for trend = 0.03) for hyperuricemia.These findings were replicated in a smaller Malaysian cohort study [67], which found that an increase in serum PTH levels was positively associated with an increase in UA levels (β = 0.165; *p* = 0.001).

Regarding PHPT, the meta-analysis by Ponvilawan et al. tried to organize the literature data by comparing UA concentrations in patients affected by PHPT and control populations [9]. The data, which came from nine highly heterogeneous studies (I^2^ = 90%), showed significantly higher UA levels in the PHPT group, with a mean difference of 65.00 μmol/L compared to the control group (95% CI: 37.74–92.25 μmol/L), equivalent to approximately 1.09 mg/dL. Furthermore, in 2010, an Israeli study evaluating the effects of parathyroidectomy on major cardiometabolic risk factors revealed a significant decrease in UA levels compared to pre-intervention, and similar data were confirmed in a recent Romanian study [68].

To support the hypothesis of a possible interaction between uric acid (UA) and parathyroid hormone (PTH), it is relevant to consider the known hyperuricemic effect of parathyroid hormone receptor agonists that are approved for the treatment of osteoporosis.

Regarding teriparatide (PTH1-34), a registration study documented an increase in the UA concentration of 13–20% during treatment with 20 μg/day of teriparatide and 20–25% with 40 μg/day, without clinical sequelae [69]. These findings were confirmed even in subsequent studies that documented a higher incidence of hyperuricemia, especially in patients with moderately impaired renal function and at a dose of 40 μg/day, without an increased incidence of gout, arthralgia or nephrolithiasis events [70]. In the ACTIVE trial of the more recent drug abaloparatide, a synthetic analog of PTH-related peptide (PTHrp), it was also observed that among patients with normal baseline UA concentrations, 25% of patients in the abaloparatide group and 6% of patients in the placebo group had at least one post-baseline concentration above the normal range. Once again, hyperuricemia was not associated with an increased incidence of adverse reactions such as gout or arthralgia compared to the placebo group [71].

However, it is interesting to note that the association with PTH is not the only one that emerged in the literature, as serum calcium levels also appear to be significantly associated with UA levels. A Chinese study in 2019 [72] utilized an approach similar to that of the aforementioned study by Hui et al., dividing a cohort of 6333 adults into quintiles, this time based on calcium levels, and in this case, they found that the probability of hyperuricemia increased progressively with the calcium quintile, reaching an OR maximum of 2.54 (IC 95%: 2.02–3.18, *p* per trend <0.001) in the highest quintile compared to the lowest. Another cross-sectional study of 8309 adolescents from the NHANES population found that each 1 mg/dL increase in the serum calcium level was associated with a 0.33 mg/dL increase in UA levels. Conversely, a 0.1 mg/dL increase in calcium levels was associated with an 8% increase in the risk of hyperuricemia [73]. A paucity of data is available in the literature regarding the association between calcium and UA levels in PHPT, but an old study by Christensson in 1977 [74] compared a population of 41 PHPT patients with a normocalcemic control population, both groups with preserved renal function. The results showed that patients with PHPT had a positive correlation between UA and calcium levels in both females (r = 0.95, *p* not reported) and males (r = 0.97, *p* not reported), whereas this correlation was not significant regarding PTH levels. In the PHPT population, as well as in the control population, no significant correlations were observed between UA and calcium levels or between UA and PTH levels after parathyroidectomy.

## 5. Possible Mechanisms of Hyperuricemia in Primary Hyperparathyroidism

To explain this association, different mechanisms have been proposed.

### 5.1. Interaction Between PTH and Uric Acid

The group of Sugimoto et al. discovered key elements about the effects of PTH on UA metabolism [12]. In vitro, the researchers studied the effects of PTH on Caco-2 intestinal cells and demonstrated that this hormone reduces ABCG2 expression on the plasma membrane without altering mRNA levels, indicating a post-transcriptional regulatory mechanism mediated by the PTH receptor. Furthermore, researchers found that in murine models affected by secondary hyperparathyroidism, high PTH levels can downregulate ABCG2 expression in renal and intestinal epithelia, resulting in reduced urate excretion and a significant increase in UA levels. The use of cinacalcet, a calcimimetic drug that can reduce PTH levels, in these animals prevented the ABCG2 reduction and urate accumulation, suggesting a potential therapeutic approach to mitigate SHPT-induced hyperuricemia.

Another complicating factor is the inverse relationship between 25(OH)and UA levels [75,76,77], and the documented suppressive effect that UA has on 1-α-hydroxylase activity [78], the enzyme responsible for the conversion of 25(OH)D to its active form 1,25(OH)_2_D.

**Figure 5:**
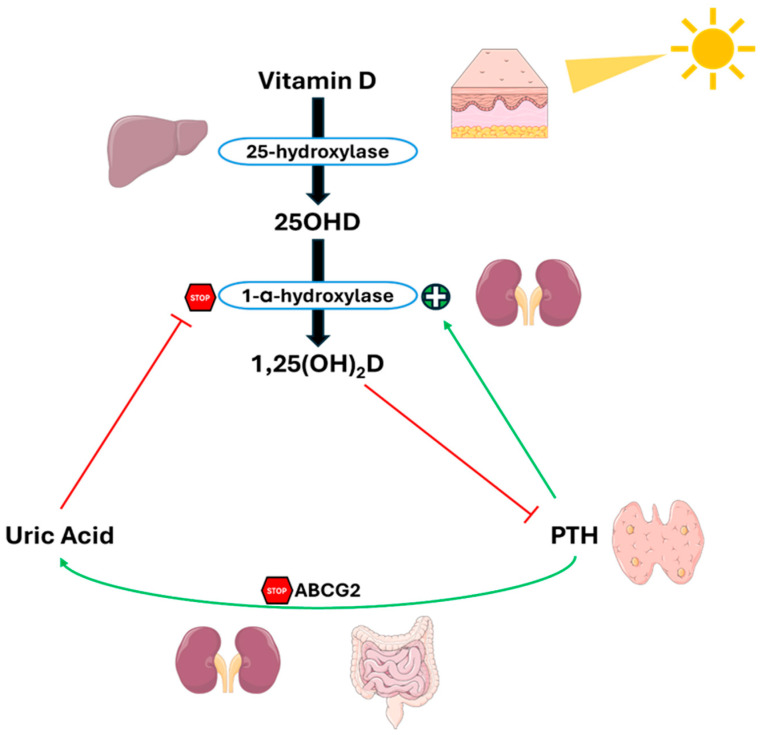
**Crosstalk between PTH, uric acid and 1,25(OH)_2_D in PHPT.** PTH reduces UA excretion by downregulating ABCG2. Elevated UA levels inhibit 1-α-hydroxylase, lowering 1,25(OH)_2_D levels and increasing PTH levels, and creating a feedback loop between hyperparathyroidism and hyperuricemia. Abbreviation: ABCG2, ATP-binding cassette super-family G member 2. Adapted from Ponvilawan et Charoenngam [79].

The same Thai group that conducted the meta-analysis tried to clarify the complex relationship between UA, PTH, and vitamin D, presenting a complex feedback system, as shown in Figure 5 [79]. In this context, PTH reduces UA excretion by suppressing ABCG2 expression. The subsequent increase in UA levels inhibits 1-α-hydroxylase, causing a reduction in 1,25(OH)_2_D levels and a consequent rise in PTH levels, thus creating a vicious cycle between hyperparathyroidism and hyperuricemia.

Although this review primarily focuses on PHPT and examines the PTH-UA relationship, it is important to consider that this interaction extends beyond PHPT. Secondary hyperparathyroidism (SHPT), which is typical in CKD patients, shares many pathological mechanisms with PHPT, including UA metabolic disruption [10]. Conversely, hyperuricemia promotes CKD progression via vascular, inflammatory, and fibrotic pathways, exacerbating mineral disturbances such as phosphate retention, hypocalcemia, and reduced vitamin D activation, thereby promoting PTH secretion and parathyroid hyperplasia and resulting in SHPT [80,81].

### 5.2. Interaction Between Serum Calcium and Uric Acid

Data on the physiopathological mechanisms of this association are less solid. It is known that the main transporters responsible for calcium excretion in the basolateral plasma membrane of the proximal tubule—PMCA (plasma membrane Ca^2+^ ATPase) and NCX (Na^+^/Ca^2+^ exchanger)—are influenced by reactive oxygen species (ROS) [82,83,84,85]. UA is also reabsorbed at the proximal tubule level by the URAT1 transporter, and it is known that intracellular ROS production is induced in tubule cells by UA [86,87,88], which could alter the functions of PMCA and NCX1 [73].

Another proposed mechanism sees hypercalcemia as the main driver, because it determines an increase in the amount of calcium delivered to the nephron, resulting in nephrogenic diabetes insipidus and reducing the ability to concentrate urine [9]. This occurs through alterations in vasopressin binding, the downregulation of aquaporin expression, and the alteration of the sodium gradient in the renal interstitium. Although today PHPT is diagnosed at much lower serum calcium levels than in the past, it is possible that even mild-to-moderate extracellular volume contraction could stimulate renal tubular reabsorption of UA [15], increasing serum UA levels.

### 5.3. Chronic Inflammation in Hyperuricemia and Hypercalcemia

It can be postulated that other plausible mechanisms may be associated with the chronic inflammatory state observed in cases of both hypercalcemia and hyperuricemia.

In particular, hyperuricemia can induce a chronic inflammatory state by remodeling the secretory cytokine profile through mechanisms mediated by MSU crystals and soluble serum UA.

MSU activates the NPL3 inflammasome [89] and the NF-κB (nuclear factor kappa-light-chain-enhancer of activated B cells) and MAPK (mitogen-activated protein kinases) pathways [90], leading to increased production of inflammatory cytokines such as interleukin-1β (IL-1β), interleukin-6 (IL-6), interleukin-8 (IL-8), and tumor necrosis factor-alpha (TNF-α) [91].

Soluble UA can also lead to increased levels of these inflammatory cytokines through the downregulation of IL-1Ra (interleukin-1 receptor antagonist) [92], the activation of the AKT-PRAS40 pathway [93], and the activation of the NF-κB pathway [94]. Several studies show that these cytokines can stimulate osteoclastic activity in different experimental and clinical contexts [95,96,97,98,99,100], potentially by increasing calcium release from bone. These cytokines can also modulate the expression of the calcium-sensing receptor (CaSR), generally in a positive manner [101,102], thus reducing calcium levels. However, in some specific conditions, researchers have verified that some of these can downregulate CaSR expression [103]. Conversely, CaSR can induce the formation of the NLRP3 inflammasome, and its activation has been associated with an increased expression of the same inflammatory cytokines that modulate its expression [104,105,106].

### 5.4. Uric Acid and Bone Turnover

One final mechanism we could hypothesize is related to bone turnover and the controversial role of UA in bone metabolism. Under conditions of increased bone turnover, such as PHTP, with higher cell catabolism, it is plausible that there could be an increase in purine release and, consequently, higher UA production. However, studies in the literature suggest an inverse relationship between UA and bone turnover markers [107,108]. This paradoxical phenomenon may be explained by the extracellular antioxidant properties of UA, which neutralizes ROS, thereby inhibiting formation and activation of osteoclastic lineage cells and resulting ultimately in reduced bone resorption [109]. However, at pathologically high levels of UA, the beneficial effects of UA appear to be counteracted by its intracellular pro-oxidative actions, which could stimulate bone resorption and suppress bone formation.

Nevertheless, these studies have been conducted in the context of postmenopausal osteoporosis, whereas the bone remodeling process in PHPT is quantitatively and qualitatively different [110,111].

## 6. Clinical Implications and Future Perspectives

The presence of a significant association between PHPT and UA provides important insights into the clinical management of these patients, who are at higher risk of kidney stones and joint manifestations, as shown by gout and pseudogout cases, as well as at increased risks of cardiovascular disease and CKD progression. In this context, monitoring UA levels, or at least determining them once at diagnosis, which is currently absent from the main PHPT management guidelines, could allow for an earlier diagnosis and potential preventive treatment of related complications.

Integrating UA into the initial evaluation of PHPT could improve the metabolic evaluation of these patients, thereby improving the cardiovascular and renal risk stratification. This could lead to more proactive management of modifiable risk factors, such as hypertension and dyslipidemia, as well as lifestyle interventions ranging from specific dietary advice to the potential use of hypouricemic drugs in selected patients, when additional risk factors, such as microalbuminuria, a reduced glomerular filtration rate, or metabolic comorbidities are present.

Studies with larger populations are necessary to evaluate the hyperuricemia prevalence in patients with different phenotypes of PHPT, for example evaluating if this association is confirmed in the normocalcemic subgroup.

Moreover, further research is necessary to elucidate the pathophysiological mechanisms underlying this association, as the current data appear to be substantiated only for the PTH-mediated mechanism, while other mechanisms remain largely hypothetical. As in cardiological and nephrological contexts, it is necessary to determine whether UA plays an active role in the development of PHPT-related complications or if it can serve as a marker of disease severity, with the potential therapeutic implications that would follow.

In terms of treatment, it would be interesting to deepen the impacts of parathyroidectomy and medical therapy on UA. Conversely, it would be useful to evaluate the efficacy of hypouricemic drugs in PHPT patients.

## 7. Conclusions

The association between primary hyperparathyroidism and uric acid, which was well-known but not much considered for many years, suggests the existence of linked pathophysiological mechanisms between mineral and purine metabolism.

It remains unclear whether uric acid actively contributes to complications in hyperparathyroidism or merely reflects underlying metabolic dysfunction. In the absence of specific recommendations, assessing uric acid levels at diagnosis—especially in patients with additional risk factors—may help prevent related complications.

Future studies could clarify the role of uric acid in primary hyperparathyroidism, improving our comprehension of the disease and opening the way to new therapeutic strategies to prevent cardiovascular, renal, and joint manifestations.

## Data Availability

No new data were created or analyzed in this study.

## Abbreviations

The following abbreviations are used in this manuscript:

PHPY	Primary hyperparathyroidism
BMD	Bone mineral density
UA	Uric acid
CKD	Chronic kidney disease
MSU	Monosodium urate
GLUT9	Glucose transporter 9
OAT1/3	Organic transporter 1/3
URAT1	Urate anion transporter 1
ABCG2	ATP-binding cassette super-family G member 2
TRPV5	Transient receptor potential cation channel subfamily V member 5
NCX1	Sodium/calcium exchanger 1
PMCA1b	Plasma membrane calcium ATPase 1b
TAL	Ascending limb of Henle’s loop
NCC	Na/Cl cotransporter
NaPi-II	Sodium-dependent phosphate transport protein 2
RANKL	Receptor activator of nuclear factor kappa-Β ligand
OPG	Osteoprotegerin
RANK	Receptor activator of nuclear factor κ B
TRPV6	Transient receptor potential vanilloid subfamily member 6
LVH	Left ventricular hypertrophy
NHANES	National Health and Nutrition Examination Survey
BMI	Body mass index
ROS	Reactive oxygen species
CaSR	Calcium-sensing receptor
NF- κB	Nuclear factor kappa-light-chain-enhancer of activated B cells
MAPK	Mitogen-activated protein kinases
IL-1β	Interleukin-1β
IL-6	Interleukin-6
IL-8	Interleukin-8
TNF-α	Tumor necrosis factor-alpha
IL-1Ra	Interleukin-1 receptor antagonist
